# Conceptual risk assessment of mosquito population modification gene-drive systems to control malaria transmission: preliminary hazards list workshops

**DOI:** 10.3389/fbioe.2023.1261123

**Published:** 2023-10-26

**Authors:** Ana Kormos, George Dimopoulos, Ethan Bier, Gregory C. Lanzaro, John M. Marshall, Anthony A. James

**Affiliations:** ^1^ Vector Genetics Laboratory, University of California, Davis, Davis, CA, United States; ^2^ W. Harry Feinstone Department of Molecular Microbiology and Immunology, Bloomberg School of Public Health, Malaria Research Institute, Johns Hopkins University, Baltimore, MD, United States; ^3^ Department of Cell and Developmental Biology, University of California, San Diego, San Diego, CA, United States; ^4^ Divisions of Epidemiology and Biostatistics, School of Public Health, University of California, Berkeley, Berkeley, CA, United States; ^5^ Departments of Microbiology and Molecular Genetics and Molecular Biology and Biochemistry, University of California, Irvine, Irvine, CA, United States

**Keywords:** genetic engineering, GEMS, gene-drive, human/animal health, invasiveness/persistence, target/nontarget organisms, evolution/stability, target product profile (TPP)

## Abstract

The field-testing and eventual adoption of genetically-engineered mosquitoes (GEMs) to control vector-borne pathogen transmission will require them meeting safety criteria specified by regulatory authorities in regions where the technology is being considered for use and other locales that might be impacted. Preliminary risk considerations by researchers and developers may be useful for planning the baseline data collection and field research used to address the anticipated safety concerns. Part of this process is to identify potential hazards (defined as the inherent ability of an entity to cause harm) and their harms, and then chart the pathways to harm and evaluate their probability as part of a risk assessment. The University of California Malaria Initiative (UCMI) participated in a series of workshops held to identify potential hazards specific to mosquito population modification strains carrying gene-drive systems coupled to anti-parasite effector genes and their use in a hypothetical island field trial. The hazards identified were placed within the broader context of previous efforts discussed in the scientific literature. Five risk areas were considered i) pathogens, infections and diseases, and the impacts of GEMs on human and animal health, ii) invasiveness and persistence of GEMs, and interactions of GEMs with target organisms, iii) interactions of GEMs with non-target organisms including horizontal gene transfer, iv) impacts of techniques used for the management of GEMs and v) evolutionary and stability considerations. A preliminary hazards list (PHL) was developed and is made available here. This PHL is useful for internal project risk evaluation and is available to regulators at prospective field sites. UCMI project scientists affirm that the subsequent processes associated with the comprehensive risk assessment for the application of this technology should be driven by the stakeholders at the proposed field site and areas that could be affected by this intervention strategy.

## Introduction

Advances in the development of genetically-engineered mosquitoes (GEMs) to control transmission of malaria parasites has stimulated work to define pathways from discovery through development to delivery (Carballar-Lejarazú and James, 2017). Identifying these pathways is work in progress and the science is outpacing community-based efforts to certify best practices. As a consequence, investigators, scientific advisory groups and potential stakeholders have offered analyses of challenges and issued guidelines for moving the science forward (Benedict et al., 2014; Wilson et al., 2015; National Academies of Sciences Engineering and Medicine, 2016; James et al., 2018). A common guiding principle is that the work be done in a step-wise, phased fashion during which specific criteria must be met before advancing from one phase to the next. The UCMI program applies a relationship-based model that goes further and argues that many of the criteria defined for each research phase should be determined by the end-user groups considering the application of the technology (Kormos et al., 2021; *see also*; National Research Council, 1983). An early framework for testing GEMs proposed by the World Health Organization (WHO) specified four phases: Phase 1 tests are discovery stages confined physically to laboratories and insectaries; Phase 2 studies move the strains to development and are carried out in small-scale physically- and/or ecologically-contained field tests; Phase 3 continues development in a series of open release trials that increase in size, length and complexity at one or more sites; and Phase 4 moves the technology to wider application as a malaria control tool in the delivery stage (James et al., 2010). Specific GEM strains are evaluated and subjected to rigorous ‘go/no-go’ criteria in each Phase. Subsequent efforts acknowledged special challenges posed by GEMs carrying gene-drive systems (National Academies of Sciences Engineering and Medicine, 2016; James et al., 2018).

As part of the phased evaluation, field-testing and eventual adoption of GEMs will require them meeting rigorous safety criteria along with demonstrations of efficacy, stability, cost-effectiveness and community acceptance. Many of the evaluation criteria are elaborated in the context of a target product profile (TPP), which lists ideal characteristics and minimally-acceptable thresholds defined specifically to assist go/no-go decision-making (Carballar-Lejarazú and James, 2017). Preliminary risk consideration is crucial in any pathway that ultimately certifies safety.

The UCMI program follows the recommended WHO guidelines and is currently in the beginning stages of Phase 2, which includes baseline data studies and evaluation at field sites in direct partnership with local scientists and stakeholders. The preliminary consideration of potential risks described here may be useful for field-site authorities in their defining a regulatory pathway and in weighing risks and benefits that should be addressed in a TPP.

Risk assessment has been characterized as a four-step process that includes hazard identification, dose-response assessment, exposure assessment and risk characterization (NRC, 2009). This approach was adopted by the US Environmental Protection Agency (EPA) and details developed on possible ways to conduct it (Norton et al., 1992; EPA, 2000; Raybould, 2006; Ankley et al., 2010; Wolt et al., 2010; Devos et al., 2019). Part of the process is to identify potential harms, pathways to harm and the hazards they pose. These terms have been defined in multiple contexts and as used here, ‘harm’ is a detrimental outcome of an event or activity and ‘hazard’ is an event, condition or activity with the potential to cause harm (Benedict et al., 2008).

A series of workshops was held to develop a preliminary hazards list (PHL) specific to some mosquito population modification strains and their use in a hypothetical island field trial. Population modification strategies seek to introduce into a target mosquito species one or more genes or alternative alleles that confer resistance or refractoriness to the parasites (Carballar-Lejarazú and James, 2017). These anti-parasite effector genes present at sufficient frequencies in the vector population should lower parasite transmission to a level that results in reduced morbidity and mortality. While no specific intervention product was defined during the workshops, the general properties of the envisioned strains are those engineered genetically to carry an autonomous gene-drive system linked to one or more genes that interfere with parasite development in the mosquito and therefore eliminate or reduce pathogen transmission by the vector to its human hosts. The engineered and introduced DNA would be similar conceptually to that of the Cas9/guide RNA (gRNA)-based, autonomous gene-drive system coupled to anti-parasite effector genes currently under development (Carballar-Lejarazú et al., 2020; Carballar-Lejarazú et al., 2023). The effector genes are constructed using endogenously derived *cis*-acting control (promoter and enhancer) DNA to determine when, where and how much of the effector-gene product is made. Specific activation of the gene-drive system is achieved using well-characterized gene promoter and control DNA sequences to express the Cas9 nuclease in the pre-meiotic germline of both male and female mosquitoes along with a ubiquitously- and constitutively-expressed gRNA (Carballar-Lejarazú et al., 2020; Carballar-Lejarazú et al., 2023). Activity of the gene-drive system results in the conversion of hemizygous gene-drive germline cells to homozygotes.

The initial field trial scenario envisages a small-scale release on an island where there is minimal human traffic and transport of goods that may harbor mosquitoes to an adjacent mainland area (Lanzaro et al., 2021). One such island nation meeting these criteria is the Democratic Republic of São Tomé and Príncipe (STP), where the UCMI program has been working since 2019. The prospective target organism is *Anopheles coluzzii*, an efficient vector of human malaria parasites. We present here a preliminary PHL relevant to an initial, island-based, field-release strategy and make it available for the next steps in charting possible pathways to harm.

## Methods

### Gene-drive nomenclature

The following nomenclature is adopted from previous work by the authors and others. Gene drive is any natural or synthetic phenomenon that results in the distortion of the inheritance ratios following meiosis in a diploid organism. Gene drive is used most-commonly to refer to circumstances in which one of a pair of alternative forms of a gene (alleles) in a heterozygous parent is inherited preferentially by its offspring (James, 2005). A gene-drive mechanism is the underlying biology that results in the observed inheritance distortion, an example of which includes the now widely used Cas9/gRNA technology (Macias et al., 2017; Bottino-Rojas and James, 2022). A gene-drive system is the final synthetic gene drive-inducing genetic construct based on one or more of the naturally occurring or synthetic mechanisms (James, 2005).

### Workshops

A series of six online workshops facilitated by the Foundation for the National Institutes of Health (FNIH) was carried out during August-September 2020. The first four included the UCMI researchers from University of California (UC) campuses (Berkeley, Davis, Irvine and San Diego) and Johns Hopkins University (JHU), and Transmission Zero, a Bill and Melinda Gates-funded initiative at Imperial College, London. Experts from the Commonwealth Scientific and Industrial Research Organisation (CSIRO) assisted FNIH personnel in guiding the participants in the process of developing and formatting a PHL and acted as guest observers. Following this, the UCMI scientists participated in two additional FNIH/CSIRO-facilitated online workshops that focused on summarizing the outcomes of the larger, previous workshops and reviewed the outcomes in the context of hypothetical hazards associated with gene-drive modified mosquitoes identified in the literature. UCMI participant expertise included working knowledge of vector transmission and molecular genetics, genetic and epidemiological modeling, mosquito population biology and genetics and community engagement. The outcomes of these efforts are presented in full in a CSIRO report available on the UCMI website (stopmalaria.org) and this contribution summarizes the development of the PHL.

### Literature review

Hypothetical GEM hazards available in the literature reviewed at the time of the workshops (2021) were derived from three sources, 1) biosafety regulations of relevant individual authorities (for example, The Royal Society, 1983; Norton et al., 1992; Environmental Protection Agency (EPA), 2000; Hayes et al., 2015; Hayes et al., 2020), 2) documents produced by respected international or national organizations including the WHO (WHO-TDR, 2014), Secretariat to the United Convention on Biological Diversity (UNEP/CBD, 2016), National Academy of Sciences, Engineering and Medicine (NASEM, 2016), European Food Safety Authority (EFSA, 2013; EFSA, 2020) and Australian Academy of Sciences (AAS, 2017) and 3) the views of individual or groups of scientists, published as proceedings of workshops or in “self-governance” documents (Higgs, 2003; Benedict et al., 2008; James et al., 2010; David et al., 2013; Roberts et al., 2017; Hayes et al., 2018; James et al., 2018; James et al., 2020; Rode et al., 2019; Teem et al., 2019; Romeis et al., 2020). All documents in the last two groups were reviewed by workshop participants and the hazards and issues identified within them discussed. Additional resources not considered or appearing after the workshops are listed following the References.

## Results and discussion

All hazards and issues identified in the literature review were categorized initially into one of the seven ‘areas of risk’ defined by EFSA: *‘1*) *persistence and invasiveness of the GM animal, including vertical gene transfer (VGT), 2*) *horizontal gene transfer, 3*) *interactions of the GM animal with target organisms, 4*) *interactions of the GM animal with non-target organisms (NTOs), 5*) *environmental impacts of the specific techniques used for the management of the GM animal, 6*) *impacts of the GM animal on biogeochemical processes and 7*) *impacts of the GM animal on human and animal health*’ (EFSA, 2013). An eighth risk area, designated ‘*Evolutionary and stability considerations*’ was added by workshop participants to accommodate issues raised in the literature that were deemed sufficiently different to warrant independent consideration. Overlaps among the hazards in the EFSA risk areas were identified and these were consolidated to produce five categories 1) pathogens, infections and diseases, and the impacts of GEMs on human and animal health, 2) invasiveness and persistence of GEM insects, and interactions of GEMs with target organisms, 3) interactions of GEMs with non-target organisms including horizontal gene transfer, 4) impacts of techniques used for the management of GEMs and 5) evolutionary and stability considerations. Hazards identified in all five areas belong to two general categories, those that arise as a consequence of the insertion and/or expression of the gene-drive system in the mosquitoes (‘off-target’ effects) and those that occur as a result of the presence and/or utilization of the GEMs (‘non-target’ effects) with some potential hazards overlapping both categories. Twenty-five potential hazards from the five aggregated risk areas were identified (Table 1) and their potential for generating harm (adverse effects) are discussed here in detail.

**TABLE 1:** Preliminary hazard list (PHL) for a gene drive- and island-based population modification field trial.

Area of risk		Potential hazard
Pathogens, Infections and Diseases and Human and Animal Health	1	Insertion and/or expression of the gene-drive system in mosquitoes or the presence and/or utilization of the genetically-engineered mosquitoes (GEMs) in the field causes an increase in the vectorial capacity or vector competence for the target pathogen (malaria parasite) or other non-target pathogens
	2	Insertion and/or expression of the gene-drive system selects for the emergence of target pathogens with increased virulence, possibly through the development of resistance to modified physiological mechanisms in the mosquito vector, resulting in a population of pathogens that may be transmitted more efficiently
	3	Insertion and/or expression of the gene-drive system causes reduced fitness in the GEMs resulting in an increase in the abundance of other disease-transmitting insects through pathways such as niche replacement or competitive release of another disease vector
	4	Presence and/or utilization of the GEMs results in the introduction of new pathogens into the receiving environment, including into areas where a non-GEM comparator is not present
	5	Insertion and/or expression of the gene-drive system causes physiological or behavioral differences in the GEMs that effect nuisance impacts, such as increased human biting rate
	6	Insertion and/or expression of the gene-drive system causes transmission of toxic or allergenic substances (related to the components of an engineered gene drive) either directly by biting, or indirectly by exposure from such substances released into the environment (for example, incidental exposure through inhalation or ingestion)
	7	Presence and/or utilization of the GEMs results in successful reduction and a prolonged period of low incidence of the parasite that may result in loss of immunity in human populations, requiring reliance on continued long-term positive effects of vector modification strategies to avoid resurgence of disease
Invasiveness and persistence of GEMs and Interactions of GEMs with target organisms	8	Insertion and/or expression of the gene-drive system causes unintentional genetic or behavioral changes that might decrease susceptibility to control (or surveillance) measures such as insecticides and attractants
	9	Insertion and/or expression of the gene-drive system causes changes in mosquito population parameters, fitness or behavior (e.g., altered larval competition or accelerated maturation) that may advantage GEMs as compared to the wild type, causing increased persistence and invasiveness, and possibly leading to the displacement of other insect species
	10	Long term gene-drive system expression results in a reduction in the efficacy of the GEM-mediated trait that may result in harm
	11	Insertion and/or expression of the gene-drive system causes changes in interactions with the target organisms arising from an altered genetic diversity of a reared GEM population that may result in harm
	12	Long-term utilization results in failure to achieve the quality or number of released GEM needed for intended vector or disease outcomes
Interactions of GEMs with non-target organisms, including horizontal gene transfer	13	Presence and/or utilization of the GEMs results in harms to insectivorous vertebrates due to toxins or allergens associated with the GEM
	14	Presence and/or utilization of the GEMs results in change in the abundance or species composition of pollinators and the pollination service they provide, or changes in other ecosystem services such as decomposition of organic matter, nutrient cycling, water regulation and purification (e.g., reduced larval consumption of algae causing levels of algae to increase and their associated toxins produced from algal bloom)
	15	Presence and/or utilization of the GEMs results in reductions in the abundance (or composition) of species of ecological, economic, cultural and/or social importance through competitive release if the GEM population is reduced, or from trophic consequences of species that rely on mosquitoes for food at specific times of the year
	16	Presence and/or utilization of the GEMs results in harm to the reproduction of non-target organisms through sterility or mutation
	17	Presence and/or utilization of the GEMs results in potential harms arising from the exchange of genetic information between GEMs and symbionts/parasites associated with them
Impacts of techniques used for the management of GEMs	18	Long-term utilization results in changes in resource usage and waste production of GEM production facilities
	19	Presence and/or utilization of the GEMs results in potential reductions in conventional vector control and results in impacts on mosquito population dynamics, humans health and the wider environment, including altered management and control measures of other (secondary) vector or pest species that arise as a consequence of the control of the primary vector or pest species
	20	Presence and/or utilization of the GEMs results in changes in land management in the receiving environment (e.g., wetland drainage, irrigation practices), exploitation of environmental resources or use of different control/recovery systems
	21	Long-term utilization results in changes in management responses to reduced efficacy of GEMs
	22	Long-term utilization results in changes in program activities at the release site related to mosquito surveillance and trapping
Evolutionary and stability considerations	23	Long-term utilization while under environmental selection results in changes of the GEM phenotype, including its marker and other expressed genes, after numerous generations of propagation
	24	Insertion and/or expression of the gene-drive system causes genetic rearrangements or other mutation at measurable rates
	25	Presence and/or utilization of the GEMs results in synergistic genetic interactions and unexpected phenotypic consequences of multiple “stacked’ transgenic modifications

### Pathogens, infections and diseases and human and animal health

*Hazard 1*: Gene-drive system insertion and expression and the use of the GEM strains in the field may impact vectorial capacity or vector competence for the target pathogen (malaria parasite) or other non-target pathogens. Pathogens, infections and diseases adversely impact both human and animal health with considerable overlap in potential hazards. An increase in vector competence or vectorial capacity could affect either vertebrate host group. Using the Ross-MacDonald model for malaria transmission as an example to predict impacts on humans, increases in human malaria incidence could result from an increase in the post-release abundance of the vector that changes the vector-to-host ratio within a defined region or an increase in GEM vectorial capacity or vector competence as compared to their wild-type counterparts (Smith et al., 2012; Rainer, et al., 2013). The hypothetical scenario of releasing a population modification strain on an island could involve release of only males at ratios between 1% and 10% of the wild-type male population. These ratios are based on the performance of gene-drive systems in anophelines in small laboratory cage trials (Gantz et al., 2015; Pham et al., 2019; Adolfi et al., 2020; Carballar-Lejarazú et al., 2020; Carballar-Lejarazú et al., 2023). Since male mosquitoes do not bite, their release should have no direct effect on the vector-host ratio as only females transmit pathogens. However, sex-separation procedures are not 100% effective and a small percentage (conservatively 5%, ≤0.5% of total mosquito population for a 1:10 release) of the released mosquitoes may be females. This in theory could lead to a temporary increase in pathogen transmission, but the magnitude of this effect could be mitigated using laboratory biosafety procedures designed to ensure that all released cohorts are free of any human or animal pathogens (population modification females are designed to be resistant to infection by the targeted pathogen). Furthermore, density-dependent larval mortality is expected to cause the population of adult female mosquitoes to equilibrate within a few reproductive cycles, although such effects are difficult to measure given the stochasticity of mosquito populations in the field.

There is currently no evidence to indicate that previous genetic modification has increased GEM vector competence for target or non-target pathogens (Pike et al., 2017). However, the release strains can be designed to mitigate this by careful selection of the gene drive-system chromosomal target locus to avoid disrupting any gene known to affect vector competence and any changes can be tested in the laboratory with parasite challenge assays. These tests will only be considered if there is an expectation that the expression of the novel genes or insertion sites of the modification impact some intrinsic feature of the competence of the mosquito for non-target pathogens. Indirect effects on vectorial capacity parameters due to changes in the microbiome of GEMs could occur but it is difficult to envision how this would lead to an increase in vectorial capacity compared to wild-type mosquitoes. Nonetheless, it would be possible to test all circulating pathogens that could be vectored by anopheline mosquitos at a given release site (see below) to compare the vector capacity of resident mosquitos to their closely-related gene-drive counterparts.

Changes in vector competence or vectorial capacity parameters for the target pathogen are unlikely as the design of the gene-drive system is to introduce anti-parasite effector genes. The principal pathogen target of the potential gene-drive system mosquitoes is the human malaria parasite, *Plasmodium falciparum*. However, there are three other significant species, *Plasmodium vivax*, *Plasmodium ovale* and *Plasmodium malariae*, that cause disease in humans and both *P. vivax* and *P. ovale* are found rarely in STP (WHO, 2022).

Anopheline mosquitoes are known to transmit pathogens that cause diseases other than malaria, therefore it is important to consider other human and animal non-target pathogens that may be present in the release site and areas into which the mosquitoes are predicted to spread, as well as any pathogens of concern identified by stakeholders or regulators. For example, mosquitoes in the *An. gambiae* complex can transmit seven other human or animal pathogens, Bwamba virus, lymphatic filariasis, Ngari virus, o’nyong virus, *Rickettsia felis*, Rift Valley fever virus and Tataguine virus (Hayes et al., 2020). Searches of a published materials database failed to identify any reports of Bwamba, Ngari, o’nyong or Tataguine viruses in STP (PubMed [nih.gov]). Lymphatic filariasis has been recorded there (transmitted also by *Culex quinquefasciatus*), as well as *R. felis* in animals (Ruiz et al., 1994; Sabatinelli et al., 1994; Fan et al., 2013; Roger et al., 2014; Tsai et al., 2020). While the arboviruses causing dengue and chikungunya fever also have been detected, these are not transmitted by anopheline mosquitoes (Sang et al., 2008; Dellagi et al., 2016; Yen et al., 2016).

*Hazard 2*: Changes in parasite virulence in the host may occur through increased transmission (greater infectivity to the host at the liver stage) or through increased disease (causing more severe and damaging illness/disease). An increase in virulence also may result from greater parasite loads (intensities of infection) in either the mosquito or human hosts or increased percentages of parasites successfully negotiating the host-specific developmental transitions. However, increases in parasite load in the context of natural environments could lead to greater fitness costs to the parasite, which should limit the degree of such hypothetical increases in parasite burden in the mosquito vector. *Plasmodium falciparum* has ∼6,000 annotated genes and the mosquito stages that are targeted by the effector genes (ookinetes, oocysts and hemocoel-stage sporozoites) in the proposed product are not involved in infecting the human host (Gardner et al., 2002). Hence, an effector-mediated selective pressure on the parasite is less unlikely to alter its interactions in the human host. Furthermore, it is possible that parasites could be selected for resistance to a single effector gene product, but this will not necessarily result in increased virulence, and therefore may not pose a hazard above the pre-existing state. It is important to note that the design proposed for the mosquitoes strains considered here involves dual effector genes targeting different aspect of parasite biology, which is expected to decrease significantly the probability of selecting effector-resistant parasites (Isaacs et al., 2011; Isaacs et al., 2012; Carballar-Lejarazú and James, 2017; Carballar-Lejarazú et al., 2023).

*Hazard 3*: Population modification strategies are designed deliberately to leave the ecosystem structure largely unchanged thereby preventing empty niches and potential population increases of competitive species while also providing on-going protection against re-establishment of unmodified mosquitoes (Carballar-Lejarazú and James, 2017). However, genetic engineering may impose fitness costs that could reduce GEM competitiveness. This could be overcome with effective gene drives so that population modification could still occur while the abundance of the wild-type (non-engineered) target population is concurrently diminished (Unckless et al., 2015).

*Hazard 4*: The introduction of new pathogens into the target environment is unlikely because the genetic modification would be introduced into the existing wild-type genetic background and released into a location with endemic wild-type targets of identical vectoring profiles. Standard insectary operating procedures can ensure that no pathogens are introduced with the released strains (Adelman, et al., 2017).

*Hazard 5*: The workshop participants did not envision any circumstances in which the introductions of the gene-drive system would impact any behavior that would result in increased biting rates.

*Hazard 6*: The mode-of-action of the leading candidate gene-drive systems does not involve the production of toxins or allergenic substances (Carballar-Lejarazú et al., 2023). None of the effector genes are expressed using salivary gland-specific gene control sequences and Cas9 transcription is controlled by a germ-line specific promoter with a high degree of stage- and tissue-specific expression (Carballar-Lejarazú et al., 2020; Terradas et al., 2022; Carballar-Lejarazú et al., 2023). While constitutively expressed or perduring transgene products could be present in the saliva, the immunogenicity of the prospective core gene-drive system products was tested in preliminary work in a demonstration at UC Davis and the results showed no differences in the bite responses in human volunteers following exposure to GEM and control unmodified mosquitoes (https://youtu.be/041t05gchUs).

*Hazard 7*: Concerns about the loss of acquired, anti-disease immunity in human hosts are not specific to the GEM strategy proposed here and are a potential hazard for any successful malaria intervention strategy, genetic or otherwise. Modelling supports the conclusion that rapidly reducing exposure to malaria parasites can reduce disease prevalence in acquired immune populations resulting in a greater disease burden in later years (Ghani et al., 2009). However, there is no evidence to date of this hazard occurring in practice with all current applied transmission-blocking and disease-mitigation technologies including insecticide treated nets, indoor residual spraying and malaria mass-drug treatments (Pryce et al., 2018; Kigozi, et al., 2020). In addition, the prospective release site islands, São Tomé and Príncipe, are hypo-endemic for malaria, which may mitigate a potential ‘rebound effect’ (Wang et al., 2022; WHO, 2022).

### Invasiveness and persistence of GEMs and interactions of GEMs with target organisms

It is important to note that the laboratory-developed GEMs do not ‘invade’ and ‘persist’. It is the gene-drive system that moves into the indigenous wild-type genetic background through mating, and its persistence is a beneficial design feature that provides a sustainable control option that potentially removes the need for continual interventions even in the face of re-introduction of wild-type mosquitoes.

*Hazard 8*: It is difficult to imagine how the insertion and/or expression of the gene-drive system components could lead to unintentional genetic or behavioral changes that would allow GEMs to evade existing control and surveillance methods. Any significant changes would be evident in the early, pre-release Phase testing and represent a ‘no-go’ decision as they would likely prevent the GEMs from being competitive with their wild-type target population and would lead to their extinction.

The probability of enhanced insecticide resistance in the GEMs as compared to wild-type mosquitoes is unlikely because the gene-drive system moves into the target population and would have resistance profiles similar to them. Concerns about a ‘hitch-hiking’ effect, where a non-native resistance genotype accompanies the gene-drive system, can be mitigated by ensuring in Phase 1 testing that the GEMs do not carry any resistance-conferring alleles. Any Cas9-induced off-target mutations to specific codons, such as those in the *voltage gated sodium channel* (*Vgsc*) gene, resulting in knock-down resistance (*kdr*) to pyrethroids (Martinez-Torres et al., 1998), would be lower than spontaneous mutation rates estimated to be 1.00 × 10^−9^/base pair/replication in *An. coluzzii* (Rashid et al., 2022). Laboratory analyses of transgenic mosquitoes carrying effector genes confirmed this conclusion and showed no changes in insecticide-resistance phenotypes (Pike et al., 2017). However, as discussed, laboratory tests for enhanced insecticide resistance may be a procedural requirement in the development of the TPP imposed by biosafety authorities when permitting the importation or release of GEMs.

*Hazard 9*: The development of multiple Cas9/gRNA-based population modification gene-drive systems and strains has informed the discussion on the impact of these newly introduced genes on the mosquitoes carrying them. Discussions of genetic loads and resulting effects on fitness have long been a part of the insect transgenesis literature and researchers in diverse disciplines (ecology, genetics, physiology and molecular biology) often have contributed differing opinions on their potential impact, based in some cases on empirical measurements (Catteruccia et al., 2003; Marrelli et al., 2006; Amenya et al., 2010; Isaacs et al., 2011; Pham et al., 2019). Evaluating gene-drive system loads and fitness costs is key in their phased development pathway (Carballar-Lejarazú et al., 2020; Carballar-Lejarazú et al., 2023). Epidemiologically meaningful descriptions of fitness can only be obtained from field trials in which GEMs are placed in the natural environment.

Transgene-imposed loads and ensuing fitness costs can result from two effects, the direct consequences of the gene-drive system integration in the genome (insertion/position effects) and those that result from the expression of the components of the system (expression effects). The insertion event is mutagenic, it disrupts the DNA at the site of integration, and depending upon where it inserts, may affect the gene into which it inserts or genes linked closely enough to be affected by changes in DNA architecture. The introduction of enhancer and promoter DNA sequences that can interact among themselves or with other nearby regulatory sequences may result in chromatin rearrangements. One notable example of an insertion effect was observed following the disruption of both copies of the *kynurenine-white* (*kh* ^*w*^) gene in the Indo-Pakistan vector, *An. Stephensi* (Gantz et al., 2015; Pham et al., 2019). The enzyme encoded by this gene has an important role in tryptophan metabolism in adult females following a blood meal and generates precursors for the formation of eye pigments. Ablations of both copies of *kh* ^*w*^ resulting from homozygous or heteroallelic combinations of gene-drive construct insertions or non-functional alleles generated by non-homologous end-joining (NHEJ) impose a large and significant fitness cost on females, who display significantly reduced survival and fecundity following a bloodmeal (Bottino-Rojas et al., 2022). In this instance, insertion of recoded *kh* coding sequences into the gene-drive cassette eliminated all detectable fitness costs of the drive element once introduced into a laboratory population (Adolfi et al., 2020).

Expression of novel gene products and ectopic, mistimed or reduced expression of host-derived gene products also may confer a load and impact fitness. For example, ablation of the mosquito FREP1 gene reduces parasite infection intensities but also imposes a high fitness cost (Dong et al., 2018). Remarkably, a naturally occurring allelic variant that also shows the anti-parasite effect does so without apparent fitness costs (Li et al., 2013; Dong et al., 2018). Furthermore, the expressed transgene products may induce a physiological imbalance or divert or interfere with resources needed for normal survival or reproduction (Terenius et al., 2008). One study showed a GEM strain with an altered mating behavior due to an effector gene-mediated change in the microbiota, and this provided a fitness advantage to the GEM (Pike et al., 2017). Comprehensive metabolomic studies offer opportunities to investigate these types of impacts (Carballar-Lejarazú et al., 2020; Horvath et al., 2021).

Empirical tests for measuring transgene loads and fitness impacts vary, but one potential significant impact would be a reduction in the mating competitiveness of the engineered mosquitoes so that natural or directed selection processes lead to loss of the introduced genes from the population. Here gene drive-based systems have an interesting competitive advantage. Modeling efforts predict that while gene-drive systems could impose a significant fitness cost on an organism, a strong inheritance bias could overcome this disadvantage (Ribeiro and Kidwell, 1994; Marshall, 2008; Carballar-Lejarazú et al., 2023). Drives can be favored if the inheritance bias compensates for the fitness penalty (Noble et al., 2018). Achieving a balance of these two characteristics (drive efficiency/fitness) is key to the development of successful population modification gene-drive systems.

A variety of life-history parameters associated with viability and vigor (such as life-stage specific mortality rates, adult longevity), fecundity (number of eggs laid, egg hatching rates) and fertility (percent of females laying eggs) and mating competitiveness are evaluated during Phase 1 testing (Carballar-Lejarazú et al., 2020; 2023). GEM fitness can be lower than that of wild-type comparators due to their relatively rapid adaptation to laboratory conditions and the reduced genetic diversity of these populations (Catteruccia et al., 2003). However, the workshop participants also noted that the microbiome and/or transcriptome of GEMs may be different from wild-type and this can influence fitness (citing differences in mating choice in laboratory studies) and noted that competitive interactions with other species are possible, most likely in the larval aquatic ecosystems. Nevertheless, any changes to the fitness of GEMs were thought likely to be modest.

**FIGURE 1:**
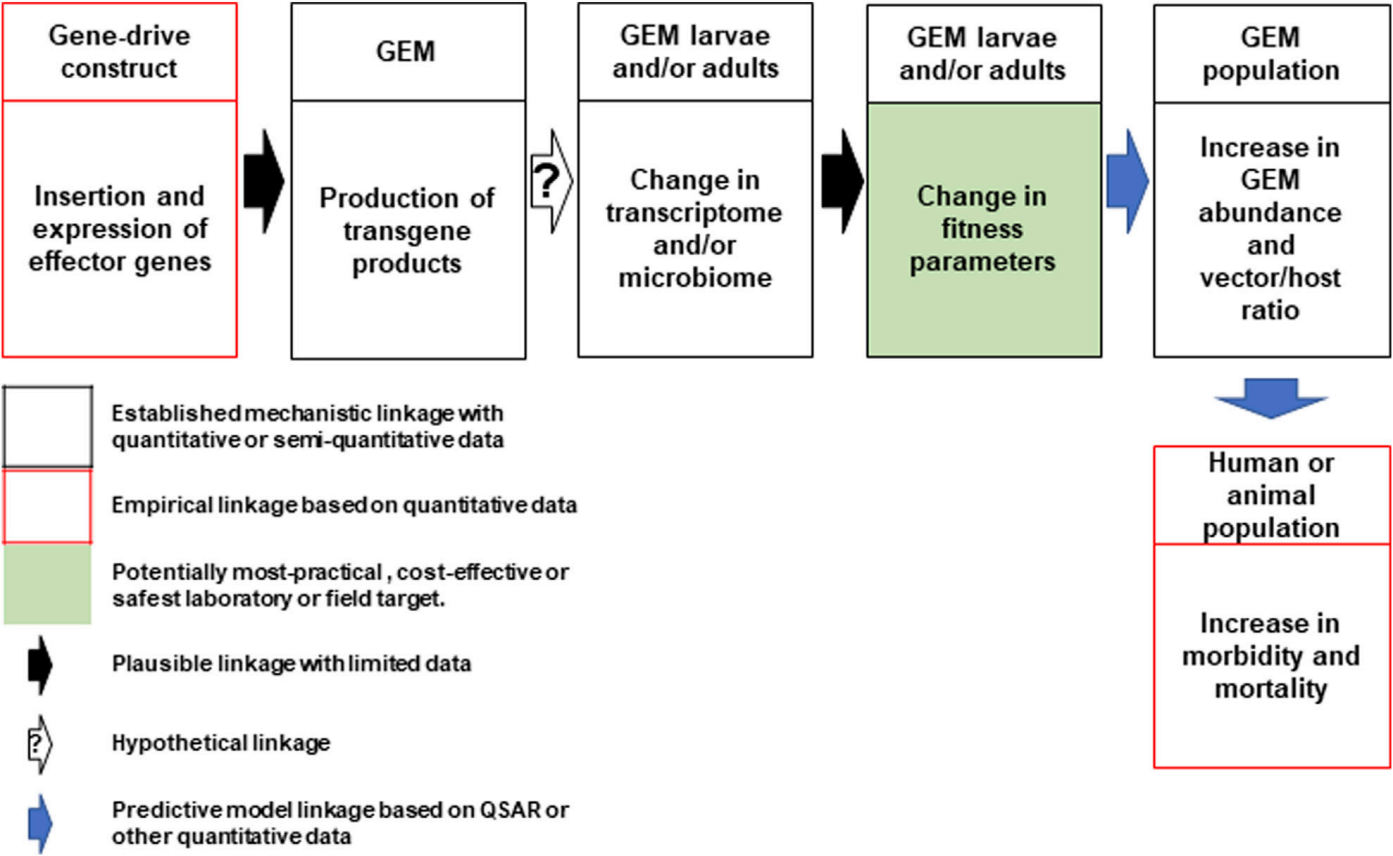
Conceptual pathway to harm for the potential hazard: increase in vectorial capacity of GEM for non-target pathogens. Following synthesis and insertion of the gene-drive construct to produce the genetically engineered mosquito (GEM; top left), transgene expression in the larval or adult stages may lead to changes in the mosquito transcriptome or microbiome leading to changes in fitness parameters (top center). This may lead to an increase in GEM vector abundance and alter the vector/host ratio (top right). This could lead to an increase in human or animal morbidity and mortality (bottom right). Linkages for each step are categorized based on an established mechanistic quantitative or qualitative data (black box) and empirical quantitative data (red box). The potential most practical, cost effective or safest laboratory or field target for evaluation is shaded in green. Plausible, hypothetical and model-based linkages are shown with black, white (with question mark) and blue arrows. QSAR are quantitative structure–activity relationship models used conceptually in evaluating drugs or chemicals but could be adapted here (Kim and Kim, 2015).

**FIGURE 2:**
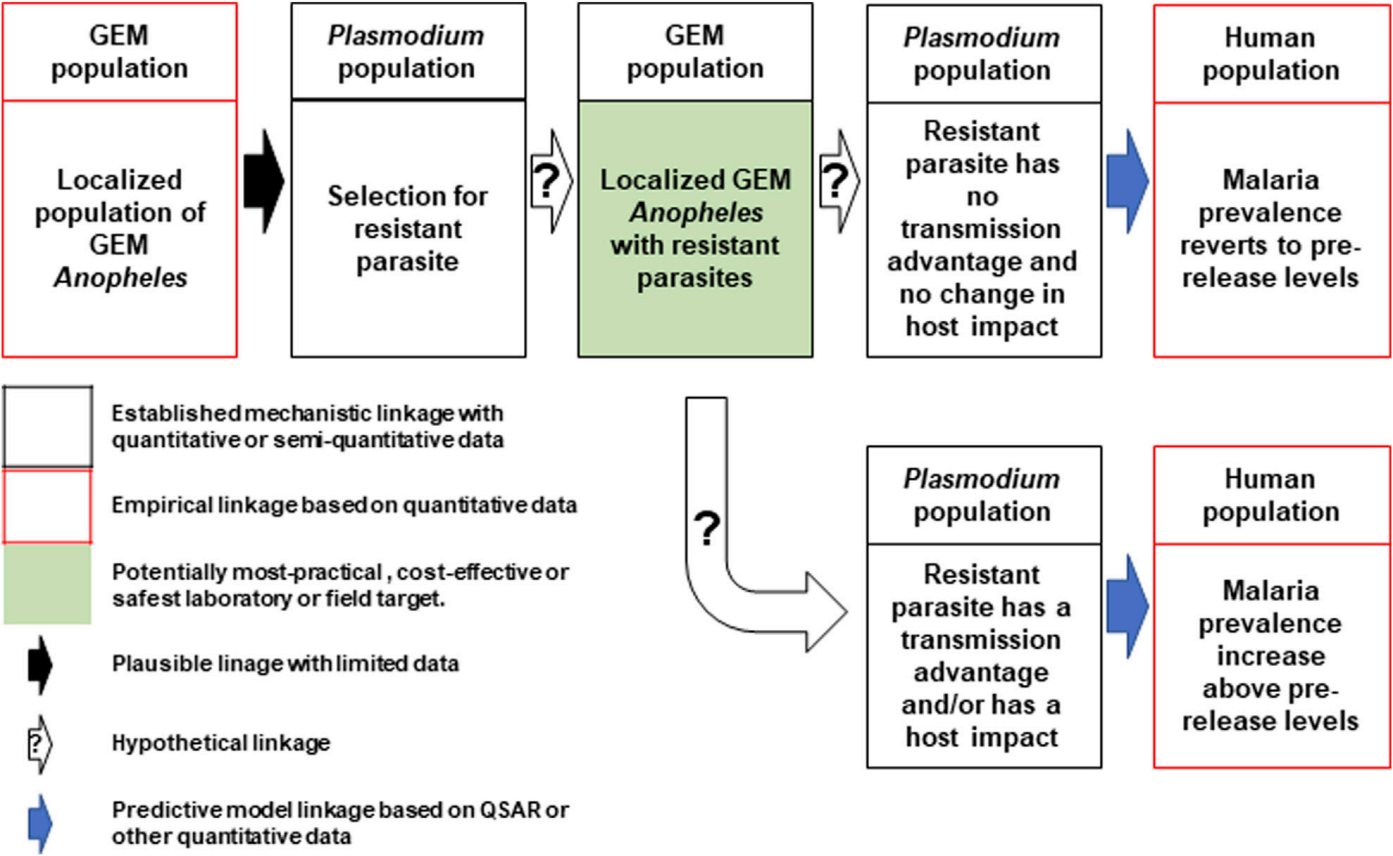
Conceptual pathway to harm for the potential hazard: increase in malaria due to emergence of target pathogens with transmission advantage. The release of the genetically engineered mosquitoes (GEM) imposes selection pressures that lead to the emergence of parasites resistant to the effector molecules (top left and center). Two outcomes are possible, the first of which produces mosquitoes with no changes in transmission dynamics and malaria prevalence reverts to pre-release levels (top right). The second outcome results in parasites with an advantage that leads to an increase in prevalence above pre-release levels (bottom right). Image notations identical to Figure 1.

In direct laboratory comparisons between source wild-type strains and their derivative gene-drive modified strains, statistically significant differences were found in a number of life-table parameters of the UCMI prospective strains but the aggregate genetic load did not affect the overall gene-drive dynamics in competitive small cage trials with 1:1 ratios of gene-drive system to wild-type males (Carballar-Lejarazú et al., 2020; Carballar-Lejarazú et al., 2023). However, the workshop participants acknowledged that these changes might lead to a small increase in the vectorial capacity of non-target pathogens by increasing the vector-to-host ratio in isolated island settings and developed a conceptual pathway to harm with plausible, hypothetical and modeled linkages supported by quantitative/semi-quantitative mechanistic and empirical data (Figure 1). The analysis also identifies the potentially most-practical, cost-effective and safest step at which to carry out laboratory or field tests. It was also noted that fitness differences could lead to an increase in the vectorial capacity of target pathogens but only when the effector gene concurrently fails, or pathogen-resistance emerges (Figure 2).

*Hazard 10*: While failure of the drive or effector molecule components could cause epidemiological conditions to revert to previous, pre-release, levels mediated by the existing wild-type mosquitoes, the participants were unable to identify any additional harms that may occur following a reduction in the efficacy of the GEM-mediated traits, aside from the already mentioned temporary bounce-back in malaria cases mentioned in *Hazard 7*.

*Hazard 11*: The genetic construct would be introduced into the wild-type genetic background prior to release and is then expected to spread into the endogenous wild-type genetic background. Introduction of any other genes responsible for laboratory-induced phenotypic behavior was considered to be unlikely based on the mechanistic biology of Cas9-based gene drives, which mobilize only the drive-system components. Hence the participants were unable to identify any additional harmful pathways due to the altered genetic diversity of the GEMs.

*Hazard 12*: No specific harms were identified arising from not having the quality or number of released GEM needed to achieve intended vector or disease outcomes. The consequences of program failure are shared by all alternative control methods. The gene-drive system design features are used to mitigate this issue. No difficulty is expected in rearing the number of transgenic mosquitoes necessary for the field trial, and failure of the construct would not result in the GEM being able to transmit malaria better than wild-type mosquitoes.

In addition to these specific hazards, the literature also notes that changes to the habitat or geographic range of the target population, including the potential for long-range, trans-boundary dispersal, and the spread of the genetic construct via gene transfer to sexually compatible species in the release area, possibly disrupting their population dynamics, may or may not lead to harmful outcomes. These areas are the subjects of on-going research.

### Interactions of GEMs with non-target organisms, including horizontal gene transfer

The literature identifies horizontal gene transfer as a potential mechanism leading to harm of non-target organisms. This mechanism was invoked in several of the pathways to harm identified by the workshop participants, so these two separate EFSA risks areas (non-target organisms/horizontal transfer) are combined here.

*Hazards 13–16* were all deemed to be implausible by the workshop participants. As noted previously, the construct is not anticipated to produce toxic or allergenic substances in the mosquito salivary glands (or elsewhere). Phase 1 trials would have precluded the release of any strain where the introduction of the construct into wild-type mosquitoes would have caused any significant change in the abundance, fitness parameters or behavior of mosquitoes. Reviews of ecosystems would be required to determine the possible non-target species that could be affected. Although few surveys exist of the total species complexity of many locations, a recent book describes thoroughly the biodiversity of the Gulf of Guinea oceanic islands that include São Tomé and Principe (Ceríaco et al., 2022).

*Hazard 17*: The literature highlights the possibility of horizontal gene transfer (HGT) to micro-organisms, noting that this could be expected *a priori* to be more likely than horizontal transfer to other insects (or eukaryotes more generally). This context raises concerns that transgenes may contain components that could confer a selective advantage to micro-organisms with which the GEMs interact; and that there may be undesirable consequences should the transgene persist in the ecosystem.

Harmful outcomes due to HGT to prokaryotes or other eukaryotes, including impacts on the reproduction of non-target organisms, or other effects following the transfer of genetic information to the symbionts and parasites associated with GEMs, were deemed to be implausible for several reasons. A recently completed intensive review of published materials found evidence for a few documented examples of HGT between prokaryotes and eukaryotes supporting the conclusion that this is rare (Wang et al., 2016). Over evolutionary time frames, HGT from *Wolbachia* to their hosts has been documented in *Aedes* mosquitoes (Klasson et al., 2009), but the team found only one report of HGT occurring in the opposite direction, that is transfer of latrotoxin genes from spiders to their bacterial endosymbionts (Zhang et al., 2012; Bing, et al., 2020). Even if the construct was transferred to prokaryotes, it is highly unlikely to be functional because the transgene promoters are exogenous, eukaryotic and highly divergent.

Similar reviews of published materials conducted previously by CSIRO identified other examples of eukaryote to prokaryote gene transfer over evolutionary time scales. A number of examples of apparent HGT from insects to *Wolbachia* have been reported and support the hypothesis that this symbiont may have adopted eukaryotic protein-coding genes (Le et al., 2012; Duplouy et al., 2013; Gabaldón, 2020).

HGT of transposable elements (TEs) among insect species has been proposed for a long time based on the non-overlap of insect evolutionary lineages with the primary structure of families of TEs (Robertson and MacLeod, 1993; Peccoud et al., 2017). However, it is only recently with the availability of many insect genome sequences and the application of analytical software that genes not related to these self-mobilizing factors have been identified (Soares de Melo and Wallau GL, 2020; Koutsovoulos et al., 2022; Li et al., 2022). These HGT events typically occur on timescales of millions of years. The mechanistic basis for HGT remains unclear although it is possible that they may ‘hitch-hike’ if integrated into the genomes of infectious agents such as viruses. Parsimonious hypotheses posit that such genes should be expected to confer a fitness advantage to those populations carrying them in order for them to reach fixation. Whether any of the gene-drive lines under investigation confer such a benefit is not known. Parasites are thought to pose some burden on the insects carrying them, but the number of actual infected mosquitoes in the wild may be too low for this to be a selective pressure in the short term. Naturally-occurring resistance to malaria parasite infection has been observed, but it has been argued that it comes with a fitness cost, and in the absence of a gene-drive system to spread it and parasite selection pressures to maintain it, it would be expected to be lost during competition with wild-type insects (Niaré et al., 2002; Voordouw et al., 2009). We anticipate that HGT from mosquitoes to any other insect of the population modification gene-drive systems currently under development would be an anomalous, low frequency event and be selected against.

*Horizontal gene transfer in the Anopheles gambiae species complex*: While this was not identified as a hazard by the workshop participants, it has come up in much of the literature addressing the extent of spread of gene-drive systems as a consequence of direct mating between species complex members. The *An. gambiae* complex consists of nine sibling species (Barron et al., 2019). Three of these species, *An. gambiae sensu strictu, An. coluzzii* and *An. arabiensis*, are major vectors of malaria, while the others are either minor vectors or non-vectors due to their localized distribution or animal feeding preferences (White et al., 2011). Experimental crosses between *An. gambiae sensu strictu* and *An. coluzzii* result in fertile, viable offspring with no obvious fitness costs in laboratory settings. Analyses of hybridization and introgression between *An. coluzzii* and *An*. *gambiae* in natural populations have demonstrated that hybrids do suffer reduced fitness (Lee et al., 2013; Hanemaaijer et al., 2018). Rare episodes in which assortative mating between the two species breaks down have been observed in the field resulting in hybridization rates >10% (Lee et al., 2013; Norris et al., 2015; Pombi et al., 2017). Higher levels have been observed on the western edge of the species’ distribution (Marsden et al., 2011; Vicente, et al., 2017). The workshop participants identified *An. gambiae sensu strictu* and *An. coluzzii* as the prospective target species, and due to the efficiency of the gene-drive systems under development, these may spread into either of the two species and impact parasite prevalence and intensities of infection (Carballar-Lejarazú et al., 2023). In two possible island field sites examined previously, only one of the species is known to occur, *An. coluzzii* in STP and *An. gambiae* s.s in the Comoros (Brunhes, 1977; Loiseau et al., 2018; Lanzaro et al., 2021). Laboratory studies confirm introgression of a gene-drive construct from an*. gambiae*/*An. coluzzii* hybrid strain into *An. coluzzii* but this may be difficult to achieve in nature due to the observed reduced fitness of hybrids (Carballar-Lejarazú et al., 2023).

Although there are reports of introgression between *An. gambiae s.s*. and *An. arabiensis*, and they mate readily under laboratory conditions, the resulting F1 males are sterile (F1 females are fertile) (Slotman et al., 2004; Slotman et. al., 2005a; Slotman et. al., 2005b). Subsequent fertility in backcrosses varies due to incompatible alleles. *Anopheles gambiae/An. arabiensis* hybrids in the field are rare (estimated at less than 0.1%), due likely to a variety of incomplete prezygotic mating barriers and selection acting against these hybrids (Slotman et al., 2004; Slotman et. al., 2005a; Slotman et. al., 2005b; Fontaine, et al., 2015; Pombi et al., 2017). Therefore, transfer of the genetic construct to *An. arabiensis* was considered highly unlikely, not necessarily harmful, and not identified as a plausible pathway to harm. Importantly, *An. arabiensis* is not present on the prospective islands being evaluated by UCMI (Brunhes, 1977; Loiseau et al., 2018).

### Impacts of techniques used for the management of GEMs

*Hazards 18–22*: The literature does not identify specific hazards in this risk area and no pathways to harm were identified by the workshop participants. The participants noted that there could be some increase in vector monitoring activities, but parasite monitoring activities would remain largely unchanged, and the anticipated reduction in insecticide use (if the trial was successful) could have positive environmental outcomes.

### Evolutionary and stability considerations

*Hazard 23*: The loss of the phenotype conferred by the modification, including its marker and other expressed genes, after numerous generations of propagation while under environmental selection is possible. The long-term activity of the drive system in the target population could impair the effector molecules through mutation or aberrant recombination. We have Phase 1 long-term cage trials in progress to try and detect this, although we acknowledge that the chance of occurrence is higher in large populations. However, any hazard imposed by this would be similar to any of the other circumstance that could arise from the lack of function of the transgene and aside from the temporary bounce-back in cases due to reduced population immunity, are not expected to produce any additional hazards above the pre-release epidemiology of malaria transmission.

**FIGURE 3:**
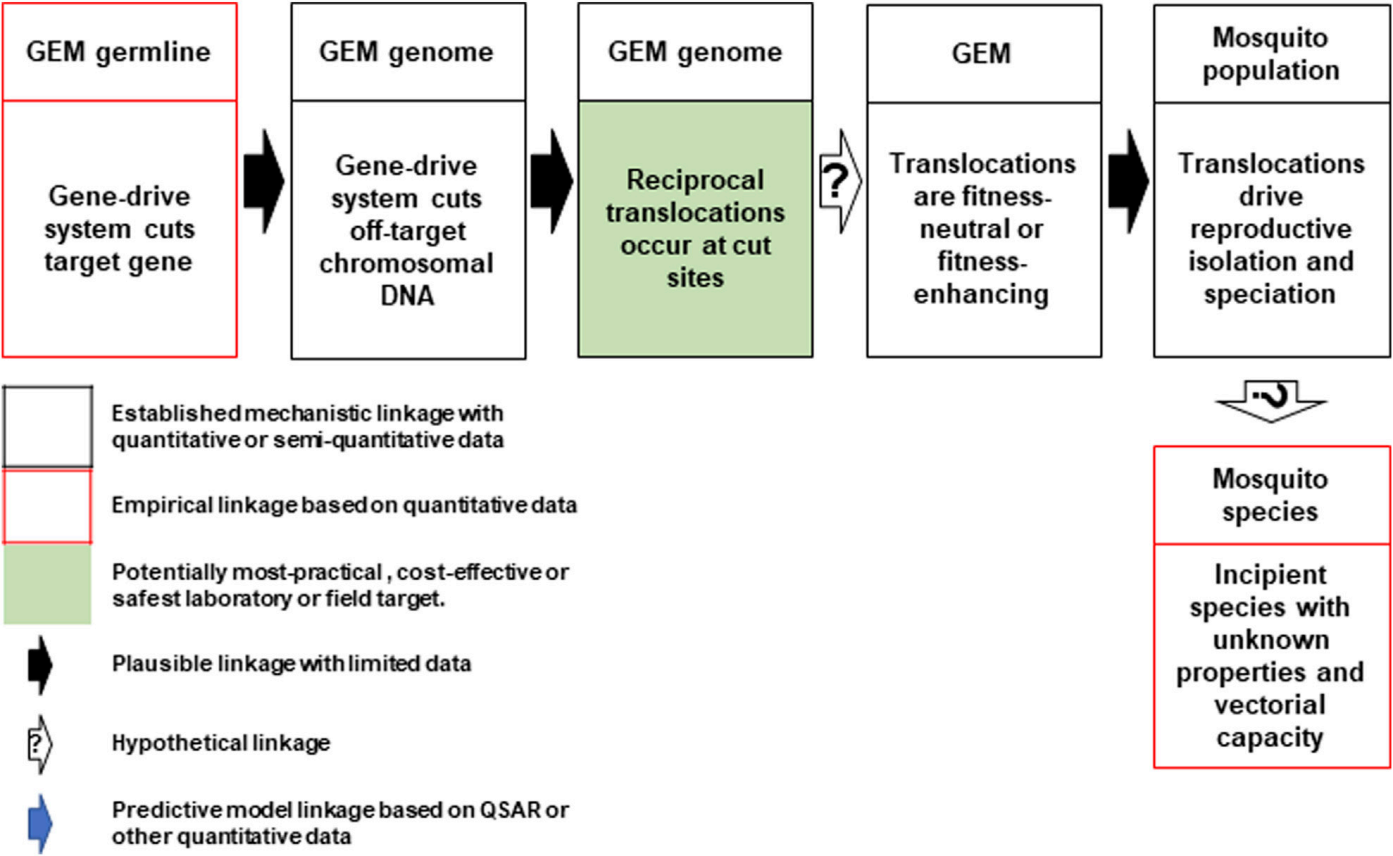
Conceptual pathway to harm for the potential hazard: emergence of new mosquito phenotypes through enhanced chromosomal translocation. The Cas9/gRNA-mediated cleavages of the target chromosome and potential off-target sites in other chromosomes results in reciprocal translocations (top left and center). If the translocations are fitness neutral or confer a reproductive advantage (top right), they may lead to reproductive isolation that results in a new mosquito species with unknown vectorial capacity for malaria parasites or other pathogens (bottom right). Image notations identical to Figure 1.

*Hazard 24*: Discussions identified a pathway to harm wherein the Cas-9 endonuclease reliably makes off-target cuts in every generation (Figure 3). NHEJ repair of off-target, Cas-9/gRNA-based double-stranded DNA breaks are known to give rise to chromosomal rearrangements such as deletions, inversions and translocations (Cho et al., 2014). Reciprocal chromosomal translocation may occur at some of these break points at rates that are higher than baseline rates associated with spontaneous mutations. These types of rearrangements can present strong barriers to gene flow between populations because they reduce recombination in heterokaryotypes, and facilitate reproductive isolation and speciation (Navarro and Barton, 2003). However, the extent to which this speciation process may lead to phenotypes with possible adverse characteristics, such as increased vectorial competence or vectorial capacity, is unknown and hypothetical at this stage. It is also unknown how the scale of such effects would compare to rearrangements events generated that lead to speciation by known genetic processes.

*Hazard 25*: Discussions of synergistic genetic interactions did not identify a specific pathway to harm but the workshop participants noted that it is theoretically possible for different constructs to interact, for example, through template switching due to shared target sequence homology. Research groups developing different gene-drive systems may need to consider ways to design them to avoid interactions with one another and incorporate such consideration into the design of any new drive system.

## Discussion

The academic literature and reports from trusted international bodies used by the workshop participants identified a number of possible hazards, associated situations and initiating events that may lead to harms on human health and environmental values following the production, release, and long-term use of GEMs. Using this information and the outcomes of a series of hazard identification workshops, our analysis highlighted three pathways to harm for a hypothetical release of genetic construct designed to make mosquitoes refractory to the *Plasmodium* parasites that cause human malaria. The presentation of these pathways emphasizes the weight of evidence that supports each step, distinguishing well-established relationships from hypothetical ones, and attempts to identify the most cost-effective, practical or safest point in the pathway to gather laboratory or field-based observations to test risk hypothesis of no harm and support subsequent risk assessment calculations. In work appearing subsequent to the efforts presented here, similar analyses were applied to gene-drive system strains for population suppression (Connolly et al., 2021; Connolly et al., 2022; Hartley et al., 2023).

Three important considerations inform this type of analysis, 1) potential hazards and the range of solutions available for mitigating them, 2) risk assessment endpoints and the choice of environmental values that are deemed to be important and worth protecting, and 3) differentiating risk hypotheses determined to be important enough to carry through to the risk-calculation stage from those that are not. It is essential that the stakeholders and communities that stand to lose or benefit from the application of the novel technologies are involved in these choices (Nelson et al., 2004; Stirling et al., 2018; Kormos, et al., 2021).

The major focus for much of the research in this field is the elimination of the on-going burden of malaria in sub-Saharan Africa (Alonso et al., 2011; WHO, 2022). We focus here on one possible control approach, the use of a genetic control technique that in theory can modify mosquito populations so that they are refractory to the major malaria-causing parasite, *P. falciparum* (Carballar-Lejarazú and James, 2017). This analysis did not canvass the opinions of scientists or stakeholders on alternative solutions, but independent studies in Africa show that community members, policymakers and regulators are generally supportive of genetic control techniques whereas scientists tend to be more skeptical (Okorie et al., 2014; Finda, et al., 2020).

The analysis here was not conducted with, or informed by, any formal stakeholder engagement activities. While the described technology is at a relatively advanced stage of discovery, and identification and consultation with relevant community groups and stakeholders is underway, the endpoint definitions and distinctions between plausible versus implausible pathways reflect the judgement, beliefs and values of only the workshop participants. While this information will inform future work, the final process for risk assessment will be determined by the appropriate authorities and communities at prospective field sites. Independent community engagement activities have taken place at African field sites and there is overlap among the concerns and issues addressed here (Finda et al., 2021). For example, concerns are expressed about the possibility of increased disease transmission and horizontal gene transfer causing harm to humans or other animals. The latter was estimated to be unlikely based on the low rates of natural HGT and the inherent design features of gene drive systems that emphasize targeting specificity, particularly within a given genus where HGT is expected to be likely. Community-expressed concerns about the genetic modification transferring to humans through biting is implausible.

A general analysis such as this is likely to be adapted by stakeholders to reflect local values and social practices. Thoughtful and intentional engagement with stakeholders should occur to ensure that the perceived risks (concerns) of local people are considered seriously and addressed. Perceived risks that developers determine unlikely may be legitimate social risks that could affect the way stakeholders and community members view the project and will influence their decision making. These considerations provide an opportunity to recalibrate and adapt the hazard analysis to new endpoints while documenting the rationale for their inclusion.

Hazard analysis exercises are early steps in risk assessment that attempt to distinguish plausible from implausible pathways and focus risk assessment on manageable numbers of potential hazards. Plausibility at this stage is the perceived probability of an event based on the experience and expertise of the participating scientists taking into consideration the best currently available data and issues relevant to stakeholders. The resulting hazard analysis exercise is a qualitative process that is expected to support the structured, specific and rational development of a quantitative risk assessment.

These early analyses are incomplete because the number of possible hazards is large and the final operational PHL used will be smaller. This applies to any risk assessment of potential alternative approaches including those that propose ‘do nothing’ or ‘business as usual’ options (Kaplan and Garrick, 1981). Those risks are well known for malaria, which causes >600,000 deaths and >2 million life-disrupting infections annually (World Health Organisation, 2022). An approach for managing this incompleteness is to consider a grouping of “other” risks that includes all the hazards not expressed (Kaplan and Garrick, 1981). The likelihood of any individual outcome within this aggregated group can be calculated by monitoring outcomes during the application of the approach, including situations in which no harms are observed, and an appropriate statistical model for the potentially relevant observations (Hayes et al., 2015). The genetically- and geographically-contained staged-release strategy is amenable to this approach by providing an opportunity for gaining operating experience in a contained and safe manner. This approach does assume that harms not considered previously would be detectable during post-release monitoring activities, but their detection is likely to be fortuitous because the outcomes would not have contributed to the post-release monitoring design.

The possibility of an incomplete hazard analysis and subsequent risk assessment cannot be eliminated for any product for which there is no previous relevant experience. This may be acceptable to stakeholders if the perceived cost/benefit balance favors the novel technology. However, it is incumbent on all those involved in the product development to do a careful, systematic and rigorous hazard analysis that addresses known concerns. Phased strategies that provide relevant field-based observations should be accompanied by hazard analysis based on checklists from the literature and be complemented by other methods designed to inform how harm might occur.

The pathways to harm analysis conducted here is an initial step in what it expected to be an iterative process of conceptual analysis and modelling. This should be followed by well-designed and co-developed limited field releases and observation, together with formal engagement and collaboration with stakeholders, and potentially complemented by additional hazard identification methodologies. The field and laboratory tests identified in this analysis also should be viewed as a minimum set that does not preclude additional tests and experiments.
